# A review of the non-bulimulid terrestrial Mollusca from the Region of Atacama, northern Chile

**DOI:** 10.3897/zookeys.398.4282

**Published:** 2014-04-04

**Authors:** Juan Francisco Araya, Ricardo Catalán

**Affiliations:** 1 Laboratorio de Invertebrados Acuáticos, Departamento de Ecología & Laboratorio de Química Inorgánica y Electroquímica, Departamento de Química, Facultad de Ciencias, Universidad de Chile. Las Palmeras 3425, Ñuñoa, Santiago. Chile; 2 Servicio Nacional de Pesca, José Santos Cifuentes 142, Caldera, Chile

**Keywords:** Land snails, Chile, Charopidae, Bothriembryontidae, Ellobiidae, Pupillidae, Strophocheilidae

## Abstract

Terrestrial mollusca are sparsely studied in Chile and, for the first time, a formal record of the diversity of land snails in northern Chile is reported. Coastal and desertic areas in the Region of Atacama, in the border of the Atacama desert and the Pacific Ocean, were surveyed with the aim to describe the presence and distribution of this poorly known fauna. Of the fourteen species recorded, the geographic distribution records for nine species are extended, and some taxa are recorded for the first time since their original descriptions. All, except one, of the fourteen terrestrial molluscan species occurring in the area are endemic to Chile; they are all terrestrial species, most of them have a restricted geographic distribution, and none of them is currently protected by law. The results reveal that the region of Atacama has one of the most diverse terrestrial snail biodiversity in Chile, ranking only after the Juan Fernandez Archipelago. Distribution records of all the studied species and a taxonomic key are also provided.

## Introduction

Terrestrial molluscs are one of the least studied invertebrate groups in Chile, the first work compiling the records of land molluscan species is still extant (Stuardo and Vega 1985). Just a few subsequent studies have reviewed genera or families (Valdovinos and Stuardo 1988, Stuardo and Vargas-Almonacid 2000) or described new species, all of them micromolluscs (Vargas-Almonacid 2000, Vargas and Stuardo 2007, Miquel and Barker 2009, Miquel and Cádiz-Lorca 2009, Miquel and Araya 2013). Studies considering species from northern Chile have been very scarce, like the work of Rehder (1945), which reviewed the subgenus *Peronaeus* and the work of Valdovinos and Stuardo (1988), reviewing the genus *Plectostylus* in Chile.

This work presents an overview, with distributions and illustrations, of all the land molluscan species found in the Region of Atacama, northern Chile. Ellobiidae species are also included, taking into account their terrestrial habitat in the country. The distribution range and a taxonomic key to all the studied taxa is also provided. The aim of this preliminary paper is thus to contribute to the knowledge of the land snail fauna in Chile.

## Methods

**Figure 1.**
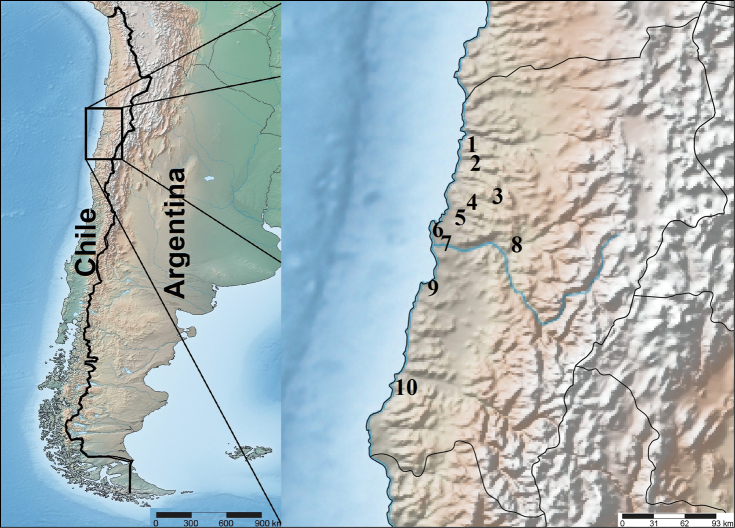
Map of sampling sites, arranged from north to south. **1** Aguas Verdes **2** Zoológico de Piedra **3** Quebrada del León **4** Plains NE Caldera **5** Caldera Bay **6** El Morro Hill **7** Chorrillos beach Area **8** Copiapó **9** Barranquilla beach Area **10** Chañaral de Aceituno.

Most of the sampling was made in the coastal desert areas around the port of Caldera (27°04'S, 70°50'W), and in specific localities in the Region of Atacama, northern Chile, during the summers of 2009 to 2012 and in August–December 2012. This region occupies the southern part of the Atacama desert and has an arid to hyper-arid climate, with low precipitation, mostly associated with the El Niño Southern Oscillation (ENSO) events. Detailed descriptions of the surveyed area, particularly of the flora and higher fauna are provided in Squeo et al. (2008). A synopsis of all the localities is given in Table 1. The surveys used a similar approach like Cowie and Robinson (2003) by also collecting litter for further sorting in the laboratory. The terminology of shell morphology is based upon Breure (1979). Original descriptions of all species were carefully reviewed, and the references included in the synonymies are mostly the ones that contained detailed descriptions or figures. Dimensions of the shells, measured with Vernier callipers (± 0.1 mm) are depicted in the Figure 1. Abbreviations used for repositories of material are: JFA-LG, private collection of the author section land Gastropoda, Santiago, Chile; MZUC, Museo de Zoología de la Universidad de Concepción, Concepción, Chile; RMNH.MOL, Naturalis Biodiversity Centre, The Netherlands, Mollusca collection; RCG, private collection of Ricardo Catalán, Sernapesca, Caldera, Chile.

**Table 1.** Sampling sites, arranged from north to south.

Locality	Coordinates/Altitude	Ecology	Ocurring species
Aguas Verdes	26°52'S, 70°48'W, 60 m	Low coastal hills with rocky outcrops, scarce vegetation.	*Plectostylus broderipii*, *Plectostylus coturnix*, *Sarnia frumentum*.
Zoológico de Piedra	26°56'S, 70°47'W, 94 m	Rocky outcrop with sparse vegetation.	*Plectostylus broderipii*.
Quebrada del León	26°57'S, 70°44'W, 378 m (Hill). 26°58'S, 70°45'W, 155 m (Plains).	Sandy plains and rocky hills with vegetation of cacti and desert bushes.	*Plectostylus broderipii*, *Stephacharopa calderaensis*.
Plains NE Caldera.	27°04'22"S, 70°49'03"W, 135 m	Coastal plain, almost no vegetation and rocky hills with scarce vegetation.	*Plectostylus broderipii*, *Plectostylus coturnix*.
Caldera Bay	27°04'S, 70°49'W, 54 m	Sandy plains with very scarce vegetation.	*Plectostylus coturnix*, *Cornu aspersum*, *Marinula pepita*, *Sarnia frumentum*.
El Morro Hill	27°08'43"S, 70°55'42"W, 194 m	Steep rocky terrain, herbs and cacti, plentiful lichen communities.	*Plectosylus coturnix*, *Pupoides (Ischnopupoides) minimus*, *Stephacharopa calderaensis*.
Chorrillos beach Area	27°09'37"S, 70°56'40"W, 64 m	Coquina cliffs and rocky outcrops.	*Plectostylus broderipii*, *Marinula pepita*.
Copiapó	27°22'00"S, 70°19'00"W, 470 m	Small mountains, very scarce vegetation.	*Plectostylus broderipii*, *Chiliborus rosaceus*.
Barranquilla beach Area	27°42'33"S, 71°01'03"W, 123 m	Sandy plains and rocky outcrops with scarce vegetation.	*Plectostylus elegans*.
Chañaral de Aceituno	29°01'35"S, 71°26'20"W, 174 m	Sandy hills with scarce vegetation.	*Chiliborus pachychilus*.

## Systematics

### Family Bothriembryontidae Iredale, 1939

#### Genus Plectostylus

Beck, 1837

##### Type species.

*Bulimus peruvianus* Bruguière, 1789, by subsequent designation (Gray 1847).

The genus is extant and distributed in Chile and Argentina, its type species is endemic to Chile. All the species have a minutely rugose, granulate or striate protoconch.

#### Plectostylus broderipii

(Sowerby I, 1832)

http://species-id.net/wiki/Plectostylus_broderipii

Figs 3.1–3.4 , Table 2

Bulinus broderipii Sowerby I, 1832: 30, figs 1,1*. *Bulimus (Plectostylus) broderipii*: Beck 1837: 58. *Bulimus broderipii*: Reeve 1849: pl. 16, fig. 97; Hidalgo 1870: 117. *Bulimus (Plectostylus) broderipi*: Pilsbry 1897: 4, pl. 6, figs 79–83. *Plectostylus broderipi*: Stuardo and Vega 1985: 135; Valdovinos and Stuardo 1988: 121, figs 86–88, pl. 3, figs 28–30, Table 3; Valdovinos 1999: 151; Neubert and Janssen 2004: 203, Taf. 13, fig. 156; Köhler 2007: 141, fig. 69; Breure and Ablett 2012: 8, figs 4A–B, 4i.

##### Material examined.

El Morro hill (27°08'43"S, 70°55'42"W) and Aguas verdes sector (26°52'S, 70°48'W), Commune of Caldera, JFA 100112, 35 specimens, RMNH.MOL.329662 (lot).

##### Diagnosis.

Shells elongate-globose, imperforate, whorls convex with a pattern of axial and spiral brownish streaks. Last whorl ample, lip simple.

##### Distribution and remarks.

From Iquique (20°30'S, 69°30'W) to Huasco (Valdovinos and Stuardo 1988). This species was moderately abundant in the area, living in sand near cacti, and in rocky outcrops.

**Table 2.** Distribution range of terrestial molluscan taxa considered in this work.

Species	Distribution	References
*Chiliborus bridgesii* (Pfeiffer, 1842)	Caleta Pajonales (27°43'S, 71°02'W) to Freirina (28°30'S, 71°04'W).	Stuardo and Vega 1985 and this study
*Chiliborus pachychilus* (Pfeiffer, 1842)	Chañaral de Aceituno (29°01'35"S, 71°26'20"W) to Coquimbo (29°57'S, 71°20'W).	Stuardo and Vega 1985 and this study
*Chiliborus rosaceus* (King & Broderip, 1831)	Copiapó (27°22'00"S, 70°19'56"W) to Chiloé Island (42°52'S, 73°49'W).	Stuardo and Vega 1985 and this study
*Cornu aspersum* (Müller, 1774)	Worldwide, in Chile from Caldera (27°04'S, 70°49'W) to Chiloé Island (42°52'S, 73°49'W).	Valdovinos 1999 and this study
*Marinula pepita* King 1832	Caldera (27°04'S, 70°49'W) to Chiloé Island (42°52'S, 73°49'W), Chile and in Lima (12°02'S, 77°01'W), Peru.	Paredes et al. 2005 and this study
*Plectostylus broderipii* (Sowerby I, 1832)	Iquique (20°30'S, 69°30'W) to Huasco (28°20'S, 71°15'W).	Valdovinos and Stuardo 1988
*Plectostylus coturnix* (Sowerby I, 1832)	El Morro hill (27°08'43"S, 70°55'42"W) to Huasco (28°20'S, 71°15'W).	Valdovinos and Stuardo 1988 and this study
*Plectostylus elegans* (Pfeiffer, 1842)	Barranquilla beach (27°21'29"S, 70°20'24"W) and Huasco (28°20'S, 71°15'W).	Valdovinos and Stuardo 1988 and this study
*Plectostylus moestai* (Dunker, 1864)	Copiapó (27°22'00"S, 70°19'56"W).	Valdovinos and Stuardo 1988 and Köhler 2007
*Plectostylus punctulifer* (Sowerby I, 1833)	Paposo (25°05'S, 70°25'W) to Huasco (28°20'S, 71°15'W).	Valdovinos and Stuardo 1988
*Plectostylus variegatus* (Pfeiffer, 1842)	Paposo (25°05'S, 70°25'W) to Lomas de Huasco (28°20'S, 71°15'W).	Valdovinos and Stuardo 1988 and this study
*Pupoides (Ischnopupoides) minimus* (Philippi, 1860)	Paposo (25°05'S, 70°25'W) to La Serena (29°54'S, 71°015 W).	Stuardo and Vargas Almonacid 2000
*Sarnia frumentum* (Petit de Saussaye, 1842)	El Callao (12°02'S, 77°08'W), Peru to Aguas Verdes (26°52'S, 70°48'W), Chile.	Paredes et al. 2005 and this study
*Stephacharopa calderaensis* Miquel & Araya, 2013	Quebrada del León (26°57'S, 70°44'W) and El Morro hill (27°08'43"S, 70°55'42"W).	Miquel and Araya 2013

#### Plectostylus coturnix

(Sowerby I, 1832)

http://species-id.net/wiki/Plectostylus_coturnix

Figs 3.5–3.9 , Table 2

Bulinus coturnix Sowerby I, 1832: 30. *Bulimus coturnix*: Hupé in Gay 1854: 102, pl. 1, fig. 4; Hidalgo 1870: 115. *Bulimulus (Plectostylus) coturnix*: Pilsbry 1897: 3, pl. 6, figs 89–92. *Plectostylus coturnix*: Breure 1979: 89; Stuardo and Vega 1985: 136; Valdovinos and Stuardo 1988: 128–129, figs 86–88. Pl. 3, figs 25–27; Valdovinos 1999: 151; Neubert and Janssen 2004: 206, Taf. 13, fig. 157; Breure and Ablett 2012: 12, figs 4C–D, 4ii. *Plectostylus broderipii*: Köhler 2007: 142, fig. 71.

##### Material examined.

El Morro hill (27°08'43"S, 70°55'42"W), Commune of Caldera, JFA 100113, 12 specimens. Hills near Vallenar (28°34'S, 70°45'W), October 2010, RCG (unnumbered), 25 specimens.

##### Diagnosis.

Shells stout, elongate-globose, with convex or very convex whorls, decorated with axial and spiral brownish streaks and spots. Last whorl very ample, lip simple, rimate umbilicus.

##### Distribution and remarks.

Huasco (28°20'S, 71°15'W) (Valdovinos and Stuardo 1988). This is the northernmost record for the species. This species is easily distinguished from *Plectostylus broderipii* due to the conspicuous rimate umbilicus, the more globose whorls, stouter shell and shorter spire.

#### Plectostylus elegans

(Pfeiffer, 1842)

http://species-id.net/wiki/Plectostylus_elegans

Figs 3.10–3.14 , Table 2

Succinea elegans Pfeiffer, 1842: 56; Pfeiffer 1852: 187. *Bulimus elegans*: Hupé in Gay 1854: 104, pl. 3, fig. 2. *Bulimulus coquimbensis Var. elegans*: Pilsbry 1897: 11, pl. 8, figs 18–22. *Plectostylus coquimbensis perelegans*: Breure 1978: 201, pl. 9, fig. 14. *Plectostylus elegans*: Breure 1979: 9; Stuardo and Vega 1985: 136; Valdovinos and Stuardo 1988: 129, figs 86–88. Pl. 3, figs 34–36. *Plectostylus perelegans*: Valdovinos 1999: 151; Neubert and Janssen 2004: 222, Taf. 13, fig. 159; *Plectostylus broderipii*: Breure and Ablett 2012: 34, figs 5E–F, 5ii. (syn. n).

##### Material examined.

Barranquilla beach (27°21'29"S, 70°20'24"W), Commune of Caldera, RCG (unnumbered), 5 specimens. Aguas verdes (26°52'S, 70°48'W), Commune of Caldera, 3 specimens. MZUC 39619 (lot).

##### Diagnosis.

Shells thin, elongate-globose, with convex and slightly shouldered whorls, decorated with axial greyish, and brownish-reddish, streaks. Last whorl very ample, lip simple, periostracum shiny and transparent.

##### Distribution and remarks.

Huasco (28°20'S, 71°15'W) (Valdovinos and Stuardo 1988). The specimens here studied constitute the northernmost record for this species. Breure and Ablett (2012) synonymized this species as *Plectostylus broderipii*. However, the shells here examined were much lighter, thinner and broader than *Plectostylus broderipii*. Shell patterns, which are contained in the thin outer shell layer, can easily differenciate *Plectostylus elegans* from *Plectostylus broderipii* in having axially marked reddish-brown lines, even in juvenile specimens. Only extensive comparative anatomy, including soft parts as well as shell morphology, would certainly help to establish its true identity.

#### Plectostylus moestai

(Dunker, 1864)

http://species-id.net/wiki/Plectostylus_moestai

Table 2

Bulimus moestai Dunker, 1864: 156. *Bulimulus (Plectostylus) moestai*: Pilsbry 1897: 6. *Plectostylus moestai*: Breure 1979: 90; Stuardo and Vega 1985: 136; Valdovinos and Stuardo 1988: 131; Köhler 2007: 142, fig. 72.

##### Material examined.

no material seen.

##### Diagnosis.

Shell subrimate, ovate-conic, thin, marked with irregular chestnut streaks. Whorls six, a little convex, apex obtuse, aperture oval, peristome simple (Valdovinos and Stuardo 1988).

##### Distribution and remarks.

Cerro Bravo, Copiapó (Pilsbry 1897). Valdovinos and Stuardo (1988), in their review of the genus, could no locate specimens of this species. Although this species has been cited for the area, searches at the type locality were unsuccessful. This may represent an extinct taxon.

#### Plectostylus punctulifer

(Sowerby I, 1833)

http://species-id.net/wiki/Plectostylus_punctulifer

Figs 3.15–3.18 , Table 2

Bulinus punctulifer Sowerby I, 1833: 36. *Bulimus punctulifer*: Reeve 1849: Pl. 16, fig. 92; Hidalgo 1870: 118.
Bulimulus (Plectostylus) punctulifer : Pilsbry 1897: 317, pl. 26, figs 67–69; Pl. 8, fig. 27; Breure 1979: 90. *Plectostylus punctulifer*: Stuardo and Vega 1985: 136; Valdovinos and Stuardo 1988: 135, figs 86–88, pl. 1, figs 1-3; Köhler 2007: 142, fig. 74. *Bulimulus (Plectostylus) punctulifer*: Breure and Ablett 2012: 33, figs 5A–B, 5i.

##### Material examined.

Fray Jorge National Park (30°40'S, 71°40'W), Region of Coquimbo, July 2006, RCG (unnumbered), 3 specimens.

##### Diagnosis.

This elongated *Plectostylus* species has a thin and somewhat fusiform shell, with an acute, long spire and five slightly convex whorls, sculptured with minute granules and growth lines. The aperture is narrow and descending, somewhat expanded in the anterior side. Periostracum is thin, opaque and yellowish.

##### Distribution and remarks.

Valdovinos and Stuardo (1988) cited this species from Paposo (25°05'S, 70°25'W) to Huasco. This species was not found in the area under current study.

#### Plectostylus variegatus

(Pfeiffer, 1842)

http://species-id.net/wiki/Plectostylus_variegatus

Figs 3.19–3.21 , Table 2

Succinea variegata Pfeiffer, 1842: 56; Pfeiffer 1843: 187. *Bulimus elegans*: Hupé in Gay 1854: 102, pl. 3, fig. 1. *Bulimulus (Plectostylus) variegatus*: Pilsbry 1897: 5, pl. 6, figs 86–88. *Plectostylus variegatus*: Breure 1978: 202, pl. 9, figs 17–18; Breure 1979: 202; Stuardo and Vega 1985: 136; Valdovinos and Stuardo 1988: 137, figs 86–88, pl. 2, figs 19–21; Neubert and Janssen 2004: 233, pl. 13, fig. 155. *Plectostylus broderipii*: Breure and Ablett 2012: 43, figs 5C–D, 5iii. (syn. n.)

##### Material examined.

Hills near Vallenar (28°34'S, 70°45'W), RCG (unnumbered), 5 specimens.

##### Diagnosis.

This relatively large species (up to 52 mm) has a thin but stout shell, with an acute, somewhat short spire and five slightly convex whorls sculptured by thin growth lines and fine spiral threads. The aperture is large, oval and slightly angulated in the columellar lip, which is completely white in its anterior part.

##### Distribution and remarks.

Valdovinos and Stuardo (1988) cited this species from Paposo (25°05'S, 70°25'W) to Lomas de Huasco (28°20'S, 71°15'W). According to Breure and Ablett (2012) this species is a subjective synonym of *Plectostylus broderipii*. The specimens here examined seem slightly different; the shells are more elongated, with a larger aperture and a more acute spire. Some specimens have rimate shells, with a pseudo-umbilicus formed by the folding of the columellar lip. These specimens have a thin, opaque, persistent and delicate brownish periostracum.

### Family Charopidae Hutton, 1884

#### Genus Stephacharopa

Miquel & Araya, 2013

##### Type species.

*Stephacharopa calderaensis* Miquel & Araya, 2013, by original designation (Miquel and Araya 2013).

The genus is extant and distributed in Chile and Argentina, its type species is restricted to the Region of Atacama, northern Chile. Protoconchs of species within the genus have 40–60 axial, smooth and low ribs.

#### Stephacharopa calderaensis

Miquel & Araya, 2013

http://species-id.net/wiki/Stephacharopa_calderaensis

Stephacharopa calderaensis Miquel & Araya, 2013: 227, figs 2–5

##### Material examined.

El Morro hills (27°8'33"S, 70°55'35"W), Commune of Caldera, August 2012, JFA 100127, 37 specimens MZUC 39613 (lot), RMNH.MOL 329670 (lot). Quebrada del León sector (26°57'S, 70°44'W), JFA 100128, 12 specimens.

##### Diagnosis.

This species has a tiny (largest specimen: 3.1 mm width), orbicular, low-spired shell, sculptured by numerous axial lamellae (about 90–95 in last whorl), with a depressed apex, a thin and brownish periostracum and an ample umbilicus. Live animals are unknown.

##### Distribution and remarks.

According to Miquel and Araya (2013) this species has a patchy distribution, having been found only in the vicinites of the port of Caldera, Region of Atacama, Chile.

### Family Ellobiidae H. & A. Adams in Pfeiffer, 1854

#### Genus Marinula

King & Broderip, 1832

##### Type species.

*Marinula pepita* King, 1832, by monotypy.

The genus is extant and distributed in South Africa, New Zealand and Chile, its type species is found from Ecuador to Chile.

#### Marinula pepita

King, 1832

http://species-id.net/wiki/Marinula_pepita

Table 2

Marinula pepita King, 1832: 344; Keen 1971: 850, fig. 849; Paredes et al. 2005: 74, fig. 8.

##### Material examined.

Caldera Bay (27°04'S, 70°49'W), Commune of Caldera, July 12 2012, JFA 100501, 21 specimens.

##### Diagnosis.

This species have small shells (up to about 11 mm), brownish or reddish in colour, higher than wider, of short spire, a large last whorl and an impressed suture. Aperture is simple, with a thin lip with a tooth in the inner external lip and three more in the columellar area. Animals are traslucent, with darker tentacles and a comparatively short foot.

##### Distribution and remarks.

This species has been cited from Coquimbo to Chiloé Island, Chile (Keen 1971), and in Lima, Peru (Paredes et al. 2005). The specimens here examined constitute the northernmost record of this species in Chile. It has been found that this species feeds on remains of birds, fishes and sea urchins (Paredes et al. 2005).

#### Genus Sarnia

H. & A. Adams in Pfeiffer, 1855.

##### Type species.

*Sarnia frumentum* Petit de Saussaye, 1842, by subsequent designation (H. A. Adams 1855).

The genus and its type species are extant and distributed in Chile and Peru.

#### Sarnia frumentum

(Petit de Saussaye, 1842)

http://species-id.net/wiki/Sarnia_frumentum

Table 2

Auricula frumentum Petit de Saussaye, 1842: 105–106; Reeve 1878, vol. 20, Auricula, pl. 4, fig. 23. *Auricula avena* Petit de Saussaye, 1842: 106; Reeve 1878, vol. 20, Auricula, pl. 4, fig. 24. *Melampus avena* Dall, 1909: 204. *Sarnia frumentum* Keen, 1971: 850, fig. 2418; Marincovich 1973: 41, figs 92, 94; Paredes et al. 2005: 70, fig. 2.

##### Material examined.

Aguas Verdes (26°52'S, 70°48'W), Commune of Caldera, August 2011, JFA 100502, 15 specimens.

##### Diagnosis.

This is one of the smallest terrestrial snails found in northern Chile. They have small (up to about 7 mm) whitish-orangish shells, of subcylindrical shape, with a simple and sharp aperture, with three plyes in the columellar side.

##### Distribution and remarks.

This species has been cited from El Callao, Peru to Chañaral, Chile (Paredes et al. 2005). This is the southernmost record for this species in Chile. It has been found that this species feeds on remains of birds, fishes and sea urchins (Paredes et al. 2005).

### Family Pupillidae Turton, 1831
Genus *Pupoides* Pfeiffer, 1854

#### Subgenus Ischnopupoides

Pilsbry, 1926

##### Type species.

*Pupa hordacea* Gabb, 1866, by original designation.

The subgenus is extant and distributed in USA, northern Mexico, Cuba and Chile, its type species is restricted to southern USA.

#### Pupoides (Ischnopupoides) minimus

(Philippi, 1860)

http://species-id.net/wiki/Pupoides_minimus

Table 2

**Figure 2.**
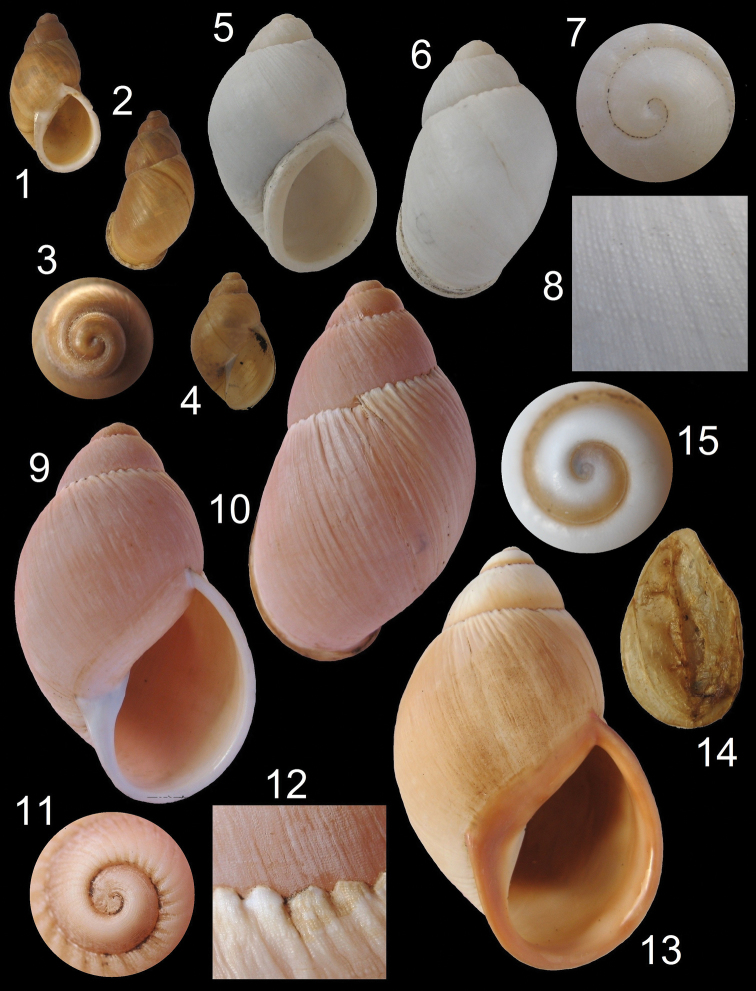
*Chiliborus* shells. *Chiliborus bridgesii*, Pajonales Bay, Province of Copiapó, 20.3 mm: **1** Ventral view**2** Dorsal view**3** Detail of protoconch **4** Juvenile shell. *Chiliborus pachychilus*, Chañaral de Aceituno, Province of Huasco, 37.3 mm: **5** Ventral view **6** Dorsal view**7** Detail of protoconch **8** Detail of sculpture. *Chiliborus rosaceus*, Los Molles, Valparaiso Region, 61.9 mm: **9** Ventral view**10** Dorsal view**11** Detail of protoconch **12** Detail of suture and sculpture**13** Ventral view of an orangish specimen, Pichidangui, Valparaiso Region, 74. 5 mm**14** Preserved epiphragm**15** Detail of protoconch.

**Figure 3.**
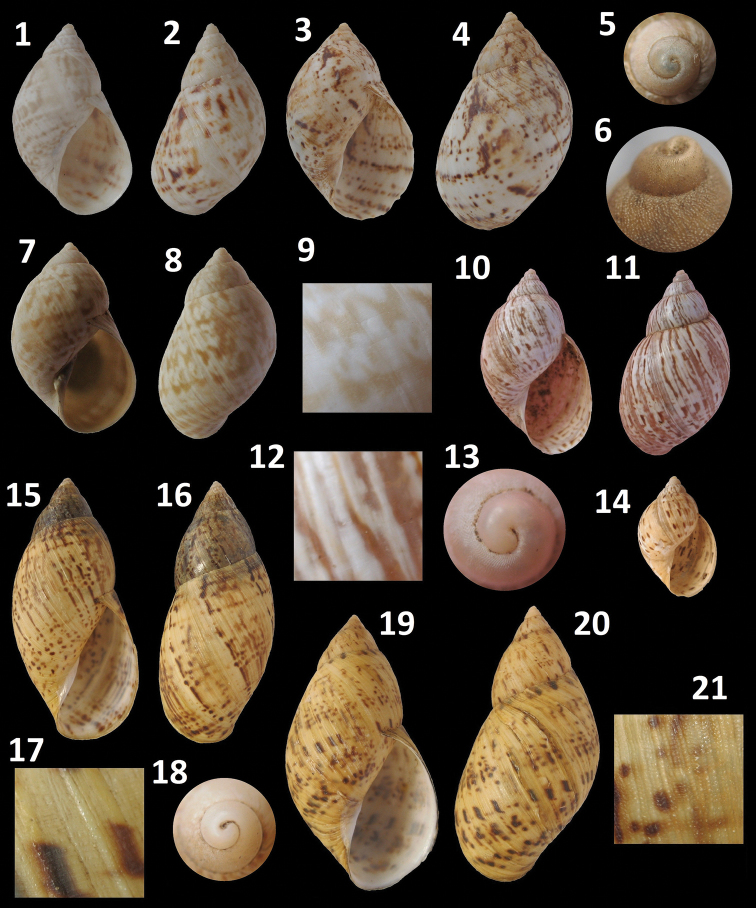
***Plectostylus* shells.** *Plectostylus broderipii*, Aguas Verdes, Commune of Caldera, 24 mm: **1** Ventral view**2** Dorsal view. El Morro hill, Commune of Caldera, 28.8 mm: **3** Ventral view**4** Dorsal view **5** Detail of protoconch*Plectostylus coturnix*, El Morro hill, Commune of Caldera, 27.5 mm: **6** Detail of protoconch**7** Ventral view**8** Dorsal view **9** Detail of sculpture. *Plectostylus elegans*, Barranquilla, Commune of Caldera, 24 mm: **10** Ventral view**11** Dorsal view **12** Detail of sculpture. **13** Detail of protoconch. **14** Juvenile shell. *Plectostylus punctulifer*, Fray Jorge National Park, Coquimbo Region, Chile, 20.2 mm: **15** Ventral view**16** Dorsal view **17** Detail of sculpture. **18** Detail of protoconch. *Plectostylus variegatus*, Vallenar, Province of Huasco, 50.5 mm.: **19** Ventral view**20** Dorsal view**21** Detail of sculpture.

Bulimus minimus Philippi, 1860: 166, Pl. 7, fig. 12a–b. *Pupoides (Ischnopupoides) minimus minimus*: Biese 1960: 133. Taf. 13, figs 1–4. *Pupoides (Ischnopupoides) minimus*: Stuardo and Vega 1985: 127; Stuardo and Vargas Almonacid 2000: 176.

##### Material.

El Morro hill (27°8'33"S, 70°55'35"W) and Zoológico de Piedra (26°56'20"S, 70°47'14"W), Commune of Caldera, September 2012 and January 2013, JFA 100126, 52 specimens, MZUC 39612 (lot), RMNH.MOL 329669 (lot).

##### Diagnosis.

This species has a tiny (up to 6 mm), whitish and elongated shell, sculptured by widely separated axial lamellae, with a small aperture and a thin and brownish periostracum.

##### Distribution and remarks.

Paposo to La Serena (Stuardo and Vargas-Almonacid 2000). Here the species seem to be narrowly distributed, with small but abundant communities found in elevated rocky areas facing the Pacific Ocean.

### Family Strophocheilidae Pilsbry, 1902

#### Genus Chiliborus

Pilsbry, 1926

##### Type species.

*Bulinus chilensis* Sowerby I, 1833, by subsequent designation (Klappenbach and Olazarri 1970).

The genus and type species are extant and endemic to Chile; protoconchs of all species of the genus have a characteristic spiral striation.

#### Chiliborus bridgesii

(Pfeiffer, 1842)

http://species-id.net/wiki/Chiliborus_bridgesii

Figs 2.1–2.4 , Table 2

Bulimus bridgesii Pfeiffer, 1842: 43; Reeve 1848: 5, *Bulimus*, pl. 19, fig. 117; Hupé in Gay 1854: 107, Malacología pl. 3, fig. 4. *Strophocheilus (Borus) bridgesii*: Pilsbry 1895: 35, pl. 2, figs 4–6. *Strophocheilus (Chiliborus) bridgesii*: Pilsbry 1926: 6. *Strophocheilus (Chiliborus) bridgesii*: Bequaert 1948: 186, pl. 13, figs 2, 3; pl. 14, fig. 3. *Chiliborus bridgesii*: Stuardo and Vega 1985: 129.

##### Material examined.

Caleta Pajonales (27°43'S, 71°02'W), Commune of Copiapó, September 2005, RCG (unnumbered), 4 specimens.

##### Diagnosis.

This species have small (up to 23 mm), pale brown, thin ovate-oblong shells, minutely sculptured by fine spiral lines, with a reflexed and delicate thin lip and a comparatively large protoconch decorated by spiral threads. This is the smallest species in the Strophocheilidae.

##### Distribution and remarks.

Freirina (28°30'S, 71°04'W) and Huasco (28°20'S, 71°15'W) (Stuardo and Vega 1985). This is the northernmost record for the species.

#### Chiliborus pachychilus

(Pfeiffer, 1842)

http://species-id.net/wiki/Chiliborus_pachychilus

Figs 2.5–2.8 , Table 2

Bulimus pachychilus Pfeiffer, 1842: 48–49. *Bulimus pachycheilus*: Reeve 1848: 5, pl. 15, fig. 87. *Strophocheilus pachychilus*: Pilsbry 1895: 35, pl. 12, figs 63–64. *Strophocheilus (Chiliborus) pachychilus*: Pilsbry 1926: 6; Bequaert 1948: 184, pl. 8, figs 2, 4. *Chiliborus pachychilus*: Stuardo and Vega 1985: 129.

##### Material examined.

Chañaral de Aceituno (29°01'35"S, 71°26'20"W), Commune of Freirina, February 2008, RCG (unnumbered), 33 specimens. MZUC 39615.

##### Diagnosis.

This species has ovate-oblong, shells (up to 39 mm), with a thin, brownish periostracum and a slightly flattened apex. They are easily distinguished by their solid and thick whitish shell and their thickened, lamellate outer lip. On magnification the surface of the shell has a rugose appearance, especially in the subsutural area, due to very fine spiral threads crossed by thin axial lines (Fig. 2.8).

##### Distribution and remarks.

Questa de Arenas, Huasco (28°20'S, 71°15'W) and Coquimbo (29°57'S, 71°20'W) (Stuardo and Vega 1985). The specimens studied here constitute the northernmost record for the species.

#### Chiliborus rosaceus

(King & Broderip I, 1831)

http://species-id.net/wiki/Chiliborus_rosaceus

Figs 2.9–2.15 , Table 2

Bulinus rosaceus King and Broderip, 1831: 341. *Bulimus (Bulimus) rosaceus*: Beck 1837: 52; Hidalgo 1870: 53. *Strophocheilus (Borus) rosaceus*: Pilsbry 1895: 33, pl. 5, fig. 26, pl. 6, figs 29–30. *Strophocheilus (Chiliborus) rosaceus*: Pilsbry 1926: 6; Bequaert 1948: 178, pl. 8, figs 2, 4. *Chiliborus rosaceus*: Stuardo and Vega 1985: 129.

##### Material examined.

Rocky hills north of Copiapo (27°21'29"S, 70°20'24"W), Commune of Copiapó, April 4 2006, JFA 100101. Pichidangui (32°08'39"S, 71°31'15"W) and Los Molles (32°13'56"S, 71°29'23"W), Region of Valparaíso, 2008, RCG (unnumbered), 9 specimens.

##### Diagnosis.

This species has large (up to 89 mm in examined specimens), brownish and elongate shells decorated with growth lines. Shells have crenulated sutures, a large protoconch and a thick lip. Animals have an orange or brownish body, with short grey tentacles.

##### Distribution and remarks.

From Huasco to Chiloé Island (42°S, 73°W) (Stuardo and Vega 1985). This is the northernmost record for the species.

### Family Helicidae Rafinesque, 1815

#### Genus Cornu

Born, 1778

##### Type species.

*Cornu copiae* Born, 1778 (= *Helix aspersa* Müller, 1774), by original designation. The genus is extant and native to Europe.

#### Cornu aspersum

(Müller, 1774)

http://species-id.net/wiki/Cornu_aspersum

Table 2

Helix (Cryptomphalus) aspersa : Stuardo and Vega 1985: 136; Valdovinos 1999: 151. *Cornu aspersum*: Landler and Nuñez 2012: 264.

##### Material examined.

Mirador de Charito sector, Caldera city (27°3'45"S, 70°50'8"W), Commune of Caldera, July 2012, JFA 100129, 2 specimens.

##### Diagnosis.

This very common species has a distinctive low-spired, brown shell with yellowish and brownish markings and four or five whorls.

##### Distribution and remarks.

According to Valdovinos (1999) this species has records in Chile from La Serena (29°54'S, 71°15'W) to the Chiloé Island, and the Juan Fernandez Archipelago (33°38'S, 78°84'W). This is the northernmost record of this species in Chile and it is the only introduced land snail species found in the area.

## Conclusions

The terrestrial molluscs found in the Region of Atacama encompasses five families: Bothriembryontidae, a Gondwanan family which in Chile is solely represented by the genus *Plectostylus*; Charopidae, a widely extended family of tiny snails; Ellobiidae, a family which includes conspicuous terrestrial species living in litoral areas, in mangroves and under rocks in salty conditions; Strophocheilidae, with conspicuously large snails and Bulimulidae, with 29 species in Chile, all in genus *Bostryx*. This last family is currently under study, with twenty three species represented in the Region of Atacama, and will be reviewed in a further work. Most of the species here considered occur in patchy distributions along the coastal desert of northern Chile, most of them with sparse records and very few have been found alive.

In summary, fourteen species of terrestrial molluscs are recorded in the Region of Atacama. All of them are ground dwellers, and only one introduced species, *Cornu aspersum*, has been found in the residential gardens of Caldera. *Chiliborus bridgesii*, *Chiliborus pachychilus*, *Chiliborus rosaceus*, *Cornu aspersum*, *Marinula pepita*, *Plectostylus coturnix*, *Plectostylus elegans*, *Plectostylus variegatus* and *Sarnia frumentum* are recorded from the Atacama region for the first time and thus they extend their distribution records in the country. Taking into account the twenty three species of Bulimulidae, which will be reviewed in another work, the number of species recorded in the region of Atacama make it one of the richest places in Chile in terms of terrestrial molluscan biodiversity. Intensive collections are needed for a further understanding of the biology and ecology of this group.

### Key for the identification of terrestrial Mollusca from the Atacama region, based on shell characters

**Table:** 

1	Shell orbicular, depressed, ample umbilicus	2
1a	Shell higher than wider, globose to turrited	3
2	Shell globose, up to 40 mm, variegated in brown-chestnut, very convex whorls, small umbilicus, ample aperture, external lip white internally	*Cornu aspersum* (Müller, 1774)
2a	Minute shell (up to 3.5 mm), convex whorls, sculptured by numerous and fine axial lamellae, ample umbilicus and flat apex	*Stephacharopa calderaensis* Miquel & Araya, 2013
3	Shell obese-ovate to elongated	4
3a	Shell very elongated or turrited, very small (up to 4.5 mm), elongate, corneous, shallow axial ribs, oval aperture	*Pupoides (Ischnopupoides) minimus* (Philippi, 1860)
4	Presence of plyes or teeth inside aperture, shells very small (up to 11 mm), last whorl very large	12
4a	Lip simple, protoconch rugose or decorated with spiral lines, shells 40 mm to 93 mm. Aperture comparatively large	5
5	Protoconch not prominent or flattened, decorated with spiral lines	6
5a	Protoconch rugose or finely striated, thin shells of medium size (up to 55 mm), whitish to yellowish in colour, variegated with brown streaks and marks, aperture very large	8
6	Shell medium sized (40 mm) to large (up to 89 mm), large protoconch, wavy suture, engrossed outer lip	7
6a	Shell small (up to 23 mm), thin, caramel-brown in colour, suture simple, reflected outer lip, yellowish thin periostracum	*Chiliborus bridgesii* (Pfeiffer, 1842)
7	Shell up to 42 mm, white to pale brown, thick, minutely sculptured by shallow spiral and axial lines, lamellated, accrescent outer lip	*Chiliborus pachychilus* (Pfeiffer, 1842)
7a	Shell large (65 mm to 89 mm), lightweight, pink-brownish, irregularly sculptured by growth marks, plicated sutures	*Chiliborus rosaceus* (King & Broderip, 1831)
8	Shell ovate-oblong or slightly fusiform, thin, no umbilical ply	9
8a	Shell stout, globose whorls, comparatively small spire, noticeable pseudo-umbilicus, aperture wider in medium part	*Plectostylus coturnix* (Sowerby I, 1832)
9	Ovate-globose shell, aperture wider in first tird of shell heigh	10
9a	Shell ovate-elongate to fusiform, yellowish to pale brownish, angulated columellar lip, surface of shell minutely granulated, yellowish periostracum	11
10	Maximum width near half of last whorl, size up to 45 mm	*Plectostylus broderipii* (Sowerby I, 1832)
10a	Shell slightly elongated, acute spire, maximum width near first third of last whorl, size up to 55 mm, delicate brownish periostracum	*Plectostylus variegatus* (Pfeiffer, 1842).
11	Shell elongate, decorated with axial brown streaks, acute spire, ovate-elongate aperture slightly flared in anterior side, yellowish periostracum	*Plectostylus punctulifer* (Sowerby I, 1833)
11a	Shell ovate, shiny, decorated with axial segmented redish-brown stripes, spire low, whorls slightly shouldered	*Plectostylus elegans* (Pfeiffer, 1842)
12	Shell brownish or reddish, short spire, impressed suture, thin lip with a tooth in the inner external lip and three more in the columellar area	*Marinula pepita* (King, 1831)
12b	Shell of subcylindrical shape, with a simple and sharp aperture and three plyes in the columellar area	*Sarnia frumentum* (Petit de la Saussaye, 1842)

## Supplementary Material

XML Treatment for Plectostylus

XML Treatment for Plectostylus broderipii

XML Treatment for Plectostylus coturnix

XML Treatment for Plectostylus elegans

XML Treatment for Plectostylus moestai

XML Treatment for Plectostylus punctulifer

XML Treatment for Plectostylus variegatus

XML Treatment for Stephacharopa

XML Treatment for Stephacharopa calderaensis

XML Treatment for Marinula

XML Treatment for Marinula pepita

XML Treatment for Sarnia

XML Treatment for Sarnia frumentum

XML Treatment for Ischnopupoides

XML Treatment for Pupoides (Ischnopupoides) minimus

XML Treatment for Chiliborus

XML Treatment for Chiliborus bridgesii

XML Treatment for Chiliborus pachychilus

XML Treatment for Chiliborus rosaceus

XML Treatment for Cornu

XML Treatment for Cornu aspersum
